# A modular ex vivo human vessel perfusion platform for studying vascular reconstruction

**DOI:** 10.1016/j.jvssci.2026.100445

**Published:** 2026-09-16

**Authors:** Chelsey T. Wood, Marc Wahab, Kentaro Noda, Marybeth Georges, Samantha Kallivretakis, Nhung Nguyen, Ibrahim Khalil, Pablo G. Sanchez, Luka Pocivavsek, Sara Gaines

**Affiliations:** a Department of Surgery, University of Chicago, Chicago, IL; b Department of Surgery, University of Illinois Chicago, Chicago, IL; c Noninvasive Vascular Imaging Laboratories, University of Chicago Medicine, Chicago, IL

**Keywords:** Ex vivo perfusion, Human vascular model, Arteriovenous fistula, Vascular remodeling, Doppler ultrasound

## Abstract

**Background:**

Vascular tissue responses to mechanical and biochemical stimuli are central to many vascular diseases and interventions, including maturation and failure of arteriovenous fistulas (AVF). Experimental systems that preserve native human vascular architecture while allowing controlled perfusion, surgical manipulation, and vascular interventions remain limited. We developed a modular ex vivo perfusion platform that supports pulsatile flow across multiple vascular configurations and enables controlled study of vessel architecture, hemodynamics, and cell biology ex vivo.

**Methods:**

Deidentified human iliac arteries and veins discarded from liver transplantation were obtained under an Institutional Review Board-approved protocol and mounted in a closed-loop circuit driven by a pulsatile blood pump. Straight arterial vessels and surgically created ex vivo AVF were perfused with endothelial cell culture medium supplemented with 1× penicillin-streptomycin, maintained at 37 °C. Flow dynamics were assessed using Doppler ultrasound. Representative vessel segments were fixed for hematoxylin and eosin, Masson trichrome, terminal deoxynucleotidyl transferase dUTP nick-end labeling (TUNEL) staining, and cleaved caspase-3 immunohistochemistry. TUNEL-positive area was quantified across whole vessel sections using Fiji (ImageJ) with Otsu automated thresholding, with manual counts of TUNEL- and cleaved caspase-3-positive nuclei performed as confirmatory analyses.

**Results:**

The platform accommodated straight arterial and surgically constructed AVF configurations during 2-hour pump perfusion. Pump settings of 40 strokes/min and 7 mL stroke volume generated pulsatile perfusion, confirmed by pressure transducers. In straight arterial runs, pulsed-wave Doppler confirmed measurable flow through the isolated human iliac artery, with velocities ranging from 4.81 to 25.18 cm/s and a mean velocity of 18.9 cm/s. In the AVF configuration, Doppler ultrasound demonstrated disturbed flow at the anastomosis, and venous outflow volume-flow rates ranged from 752.1 to 1442.5 mL/min, exceeding the 600 mL/min threshold commonly used to define functional fistula flow. Histology demonstrated close arteriovenous apposition in the constructed fistula. Pre- and postperfusion TUNEL quantification showed no increase in TUNEL-positive area after 2-hour perfusion, whereas in a separate time-course analysis, TUNEL-positive area increased with longer intervals from vessel acquisition to fixation.

**Conclusions:**

We describe a modular human vessel perfusion platform that supports pulsatile flow and clinically relevant AVF conditions ex vivo. Histologic assessment demonstrated preserved vessel architecture and no increase in apoptotic burden after short-term pump perfusion. This system provides a biologically viable human tissue platform for studying mechanical, biochemical, and device-related contributors to vascular reconstruction.

**Clinical relevance:**

Pathologic vascular remodeling contributes to critical vascular conditions, including peripheral arterial disease, adverse surgical reconstruction outcomes, restenosis, and arteriovenous fistula failure. Current models often lack intact adult human tissue under controlled flow. This modular ex vivo perfusion platform preserves native vessel architecture while supporting pulsatile perfusion, surgical manipulation, Doppler ultrasound assessment, and histologic analysis. By enabling controlled study of adult human iliac arteries and veins across multiple configurations, this system may support future investigation of vascular remodeling mechanisms, device testing, therapeutic delivery, and clinically relevant hemodynamic responses before translation to patient care.

**Article Highlights**

• **Type of Research:** Experimental ex vivo human vessel perfusion platform study
• **Key Findings:** A modular perfusion circuit supported pulsatile flow through discarded adult human iliac arteries and surgically constructed arteriovenous fistulas (AVF). Doppler ultrasound confirmed measurable arterial flow and AVF outflow above clinically relevant flow thresholds, while histologic assessment showed preserved vessel architecture and no increase in apoptotic burden after short-term perfusion.
• **Take Home Message:** This modular ex vivo human vessel perfusion platform provides a flexible model for studying vascular remodeling, AVF hemodynamics, device testing, and therapeutic delivery in intact adult human vascular tissue.

Pathologic vascular remodeling is central to many vascular diseases and intervention outcomes, including peripheral arterial disease, postintervention restenosis, maladaptive remodeling after surgical reconstruction, and arteriovenous fistula (AVF) failure.^1^^,^^2^ These processes are shaped by dynamic changes in vessel architecture, hemodynamic forces, endothelial function, and smooth muscle cell behavior.^1^^,^^3^ However, experimental systems for studying acute vascular tissue responses in intact human vessels under controlled flow remain limited. Static cell culture lacks native vessel architecture and physiologic flow, and animal models do not fully reproduce human vascular biology. Clinical studies provide essential insight into human disease, but generally do not permit controlled mechanistic manipulation or detailed tissue-level assessment under defined experimental conditions.

Attempts to bridge this gap have included umbilical or porcine vessel perfusion platforms to study human vascular tissue under flow.^4^, ^5^, ^6^ These models have demonstrated the value of maintaining native vascular architecture under controlled perfusion conditions for endothelial and therapeutic studies.^7^^,^^8^ However, additional platforms are needed that use adult human vascular tissue and support surgical manipulation, modular configurations, and clinically relevant vascular reconstructions. Building on this concept, we developed a modular platform that enables controlled perfusion of discarded human iliac vessels across multiple experimental configurations, including straight arterial perfusion and surgically constructed AVF. This platform provides an adjustable ex vivo system for studying acute vascular tissue responses to mechanical, biochemical, and device-related interventions.

## Methods

The overall experimental workflow included vessel acquisition, platform configuration, pump perfusion, ultrasound assessment, and histologic/Fiji (ImageJ) analysis (Fig 1, *A*).

**Fig 1:**
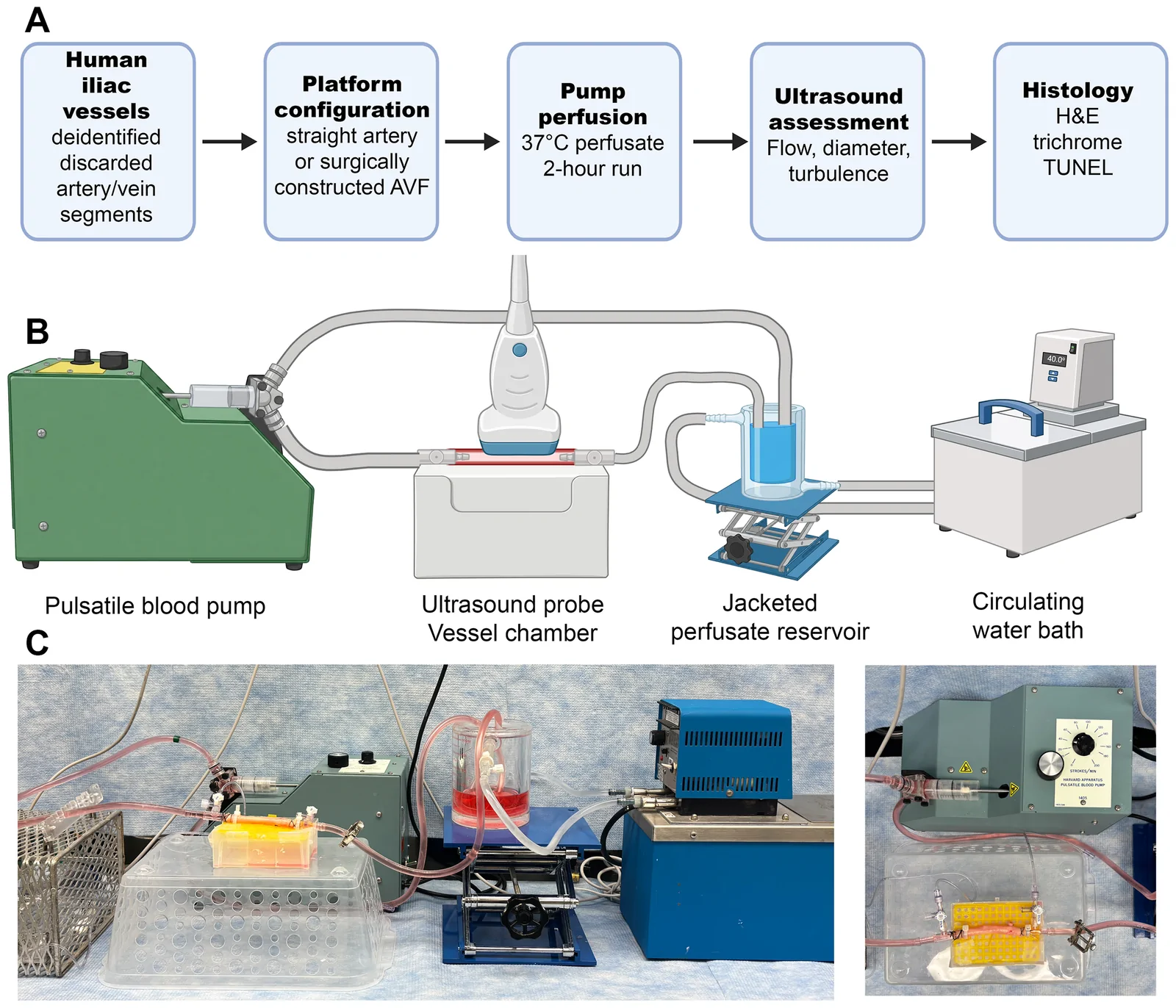
Modular ex vivo human iliac vessel perfusion platform and experimental workflow. **A,** Experimental workflow following acquisition of deidentified human iliac vessels, including platform configuration, 2-hour pump perfusion at 37 °C, ultrasound assessment, and histologic/Fiji (ImageJ) analysis. **B,** Schematic of the ex vivo perfusion system, including a pulsatile blood pump (Harvard Apparatus, model 1405), tubing, and vessel chamber, ultrasound probe, jacketed perfusate reservoir, and circulating water bath for temperature control. **C,** Representative photographs of the experimental setup. Vessels were connected to the pump circuit using ¼-inch tubing and adaptors. Inflow and outflow conditions were adjusted using clamps and a laboratory jack, and pressure transducers could be attached on either side of the vessel to measure pressure gradients across the perfused segment. *AVF*, Arteriovenous fistula; *H&E*, hematoxylin and eosin; *TUNEL*, terminal deoxynucleotidyl transferase dUTP nick-end labeling.

### Human vessel acquisition and preservation

Human iliac arteries and veins from organ donors were originally procured for transplantation, but were ultimately unused and discarded. These vessels were obtained for research under Institutional Review Board approval at the University of Chicago (IRB26-0141) and a material transfer agreement with the local organ procurement organization. National transplant policy specifies that donor vessels should typically be stored for no more than 14 days after procurement, after which unused vessels are discarded.^9^ Vessel samples were preserved in normal saline at 4 °C until experimentation. Vessels were excluded for visible damage, excessive calcification, thrombosis, contamination, or inability to be cannulated. Artery diameters ranged from 0.8 to 1.2 cm and vein diameters from 0.9 to 1.5 cm (Table).

**Table:** Summary of ex vivo human iliac vessel experiments

Experimental group	Vessel diameter, cm	Experimental replicates	Ultrasound assessment	Histologic staining	Purpose
Fixation time-course (days 3-26)	Artery: 0.8-1.2 Vein: 0.9-1.4	Artery and vein samples fixed at days 3, 7, 14, and 26	Not performed	TUNEL	Assess vessel quality and apoptotic burden across time from acquisition to histology
Straight arterial perfusion	Artery: 0.8-1.1 (no vein)	5 runs (1 artery per run)	Performed in 2/5 runs	TUNEL, Masson trichrome, cleaved caspase-3	Evaluate single-vessel platform configuration, Doppler-detectable flow, perfusion pressure monitoring, and histologic viability after perfusion
Arteriovenous fistula (AVF) perfusion	Artery: 0.8-1.1 Vein: 1.2-1.5	3 runs (1 artery + 1 vein per run)	Performed in 2/3 runs	TUNEL, Masson trichrome, cleaved caspase-3	Evaluate feasibility of surgically constructed ex vivo AVF creation, Doppler flow assessment, perfusion pressure monitoring, and histologic characterization

*TUNEL*, Terminal deoxynucleotidyl transferase dUTP nick-end labeling.

### Perfusion platform setup

Vessels were mounted in a closed-loop perfusion circuit driven by a pulsatile blood pump (Model 1405; Harvard Apparatus; Fig 1, *B* and *C*). Quarter-inch tubing and adaptors were introduced at each end of the artery or AVF and secured with silk ties in a flow tray. The pump was operated at 40 strokes/min with a 7 mL stroke volume. Inflow and outflow conditions were adjusted using Hoffman clamps and the adjustment of reservoir height. Hoffman clamps at either end of the circuit were used to regulate pressure, targeting physiologic pressures of approximately 110 to 130 mmHg systolic and 70 to 90 mmHg diastolic. Blood pressure transducers (MLT0670; ADInstruments Inc) were positioned proximal and distal to the vessel to measure pressure gradients across the perfused segment. Pressure signals were acquired using a PowerLab 16/35 data acquisition system and Quad Bridge Amplifier (ML112A; ADInstruments Inc) and recorded using PowerChart software (version 7; ADInstruments Inc). The perfusate, composed of -Dulbecco’s Modified Eagle Medium/Nutrient Mixture F-12 + GlutaMAX medium (Gibco, Thermo Fisher Scientific) supplemented with 1× penicillin-streptomycin (GenClone, Genesee Scientific), was maintained at 37 °C throughout each experiment using a jacketed reservoir and circulating water. Each perfusion trial was conducted for 2 hours. The perfusion duration was selected to assess short-term vessel viability and acute responses under controlled flow conditions rather than longer-term vascular remodeling.

### Experimental groups

The experimental design included three groups of human iliac vessel studies, summarized in Table. The straight arterial perfusion group comprised five perfusion runs, each using an iliac artery from a separate donor (n = 5 arteries from five donors), and was used to evaluate the single-vessel configuration. The AVF perfusion group comprised three surgically constructed AVFs, each using an iliac artery and vein from a separate donor (n = 3 AVFs from three donors), and was used to evaluate the feasibility of creating an ex vivo AVF. A vessel-age fixation group, using one artery and one vein fixed at 3, 7, 14, and 26 days after procurement (n = 4 arteries and four veins from four donors), was used to assess tissue quality and apoptotic burden across time from acquisition to histology. A run denotes a single ex vivo perfusion circuit.

### AVF construction

Ex vivo AVFs were created using a standard end-to-side anastomotic technique with 5-0 Prolene suture. The completed AVF was secured to the perfusion circuit using 2-0 silk ties and inspected for leaks before initiation of pump perfusion.

### Ultrasound assessment

Ultrasound images were acquired with a Mindray Resona 7 ultrasound system using L15-3WU and L9-3U linear-array transducers in transverse and longitudinal planes. Pulsed-wave Doppler was used to assess flow within the perfused vessel lumen in straight arterial and AVF configurations. When available, volumetric flow rate, peak systolic velocity, end-diastolic velocity, turbulence or color Doppler aliasing, vessel diameter, and proximal/distal pressure measurements were recorded. Measurements were performed by a registered vascular technologist using consistent acquisition parameters.

### Histology and Fiji (ImageJ) quantification

Tissue samples were fixed in formalin for 36 to 48 hours. Vessels were sectioned at 5 μm. Vessel outer diameter was measured on histologic cross-sections. Sections were stained with hematoxylin and eosin, Masson trichrome, and terminal deoxynucleotidyl transferase dUTP nick-end labeling (TUNEL) by the University of Chicago Human Tissue Resource Center. Slides were scanned using a Nikon Ti2 microscope at ×10 magnification. Whole-vessel TUNEL-stained sections were quantified in their entirety rather than from selected fields of view. TUNEL-positive area was quantified in Fiji (ImageJ) using color deconvolution followed by Otsu automated thresholding. Tissue regions of interest were generated to include the vessel wall while excluding luminal and background spaces, and TUNEL-positive area was expressed as a percentage of the tissue regions of interest. To validate the whole-section TUNEL analysis, three randomly selected ×20 fields of view were analyzed from each paired pre- and postperfusion specimen. Manual counts of TUNEL-positive and total nuclei were performed within each field, and results were expressed as the percentage of TUNEL-positive nuclei. Adjacent sections stained for cleaved caspase-3 were analyzed using the same sampling strategy, and apoptotic nuclei were manually counted as a confirmatory assessment of apoptosis.

## Results

### Platform configuration and experimental groups

The platform accommodated both straight arterial and surgically constructed AVF configurations (Fig 2). Straight iliac artery segments were connected directly to the perfusion circuit for single-vessel perfusion (Fig 2, *A*). For AVF experiments, human iliac vein was anastomosed end-to-side to human iliac artery and then secured within the perfusion circuit (Fig 2, *B*). Pump settings of 40 strokes/min and 7 mL stroke volume generated pulsatile perfusion, with pulsatility confirmed by inflow/outflow pressure measurements. Both straight-vessel and AVF configurations remained intact throughout perfusion, with minimal leakage or gross changes.

**Fig 2:**
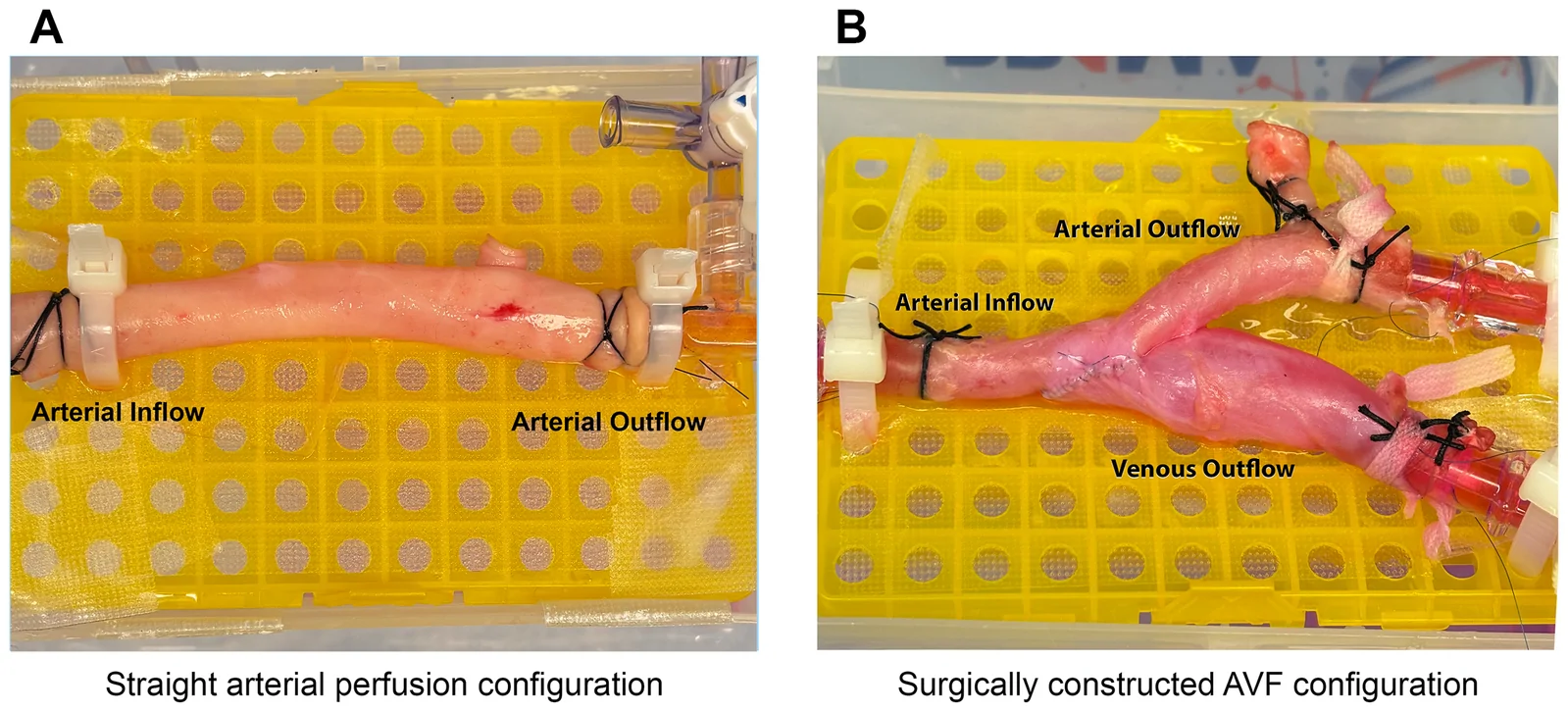
Straight-vessel and arteriovenous fistula (*AVF*) configurations in the ex vivo perfusion platform. **A,** Straight arterial configuration with the iliac artery connected to the perfusion circuit for single-vessel perfusion. **B,** Surgically constructed ex vivo AVF configuration created using a standard end-to-side anastomotic technique with 5-0 Prolene suture before initiation of perfusion. The AVF was secured to the perfusion circuit using silk ties.

### Doppler ultrasound and hemodynamic assessment of ex vivo straight vessel and AVF flow

Blood pressure transducers positioned proximal and distal to the vessel confirmed pulsatile perfusion, with systolic pressures of approximately 110 to 130 mmHg and subphysiologic diastolic pressures.

In the straight arterial configuration, pulsed-wave Doppler ultrasound confirmed measurable flow through the isolated human iliac artery, with recorded point velocities ranging from 4.81 to 25.18 cm/s and a mean velocity of 18.9 cm/s across the two runs in which Doppler was performed. Volume-flow measurements were not obtained for the straight-vessel configuration.

In the AVF configuration, brightness-mode ultrasound demonstrated the arterial inflow, anastomosis, and venous outflow (Fig 3, *A*). Doppler ultrasound demonstrated flow disturbance at the anastomosis, with disordered directional arrows consistent with turbulent AVF hemodynamics (Fig 3, *B*). Spectral Doppler assessment of the venous outflow demonstrated volume-flow rates ranging from 752.1 to 1442.5 mL/min, with a mean measured volume-flow rate of 1091.5 mL/min across the two AVF runs in which Doppler was performed (Fig 3, *C*).

**Fig 3:**
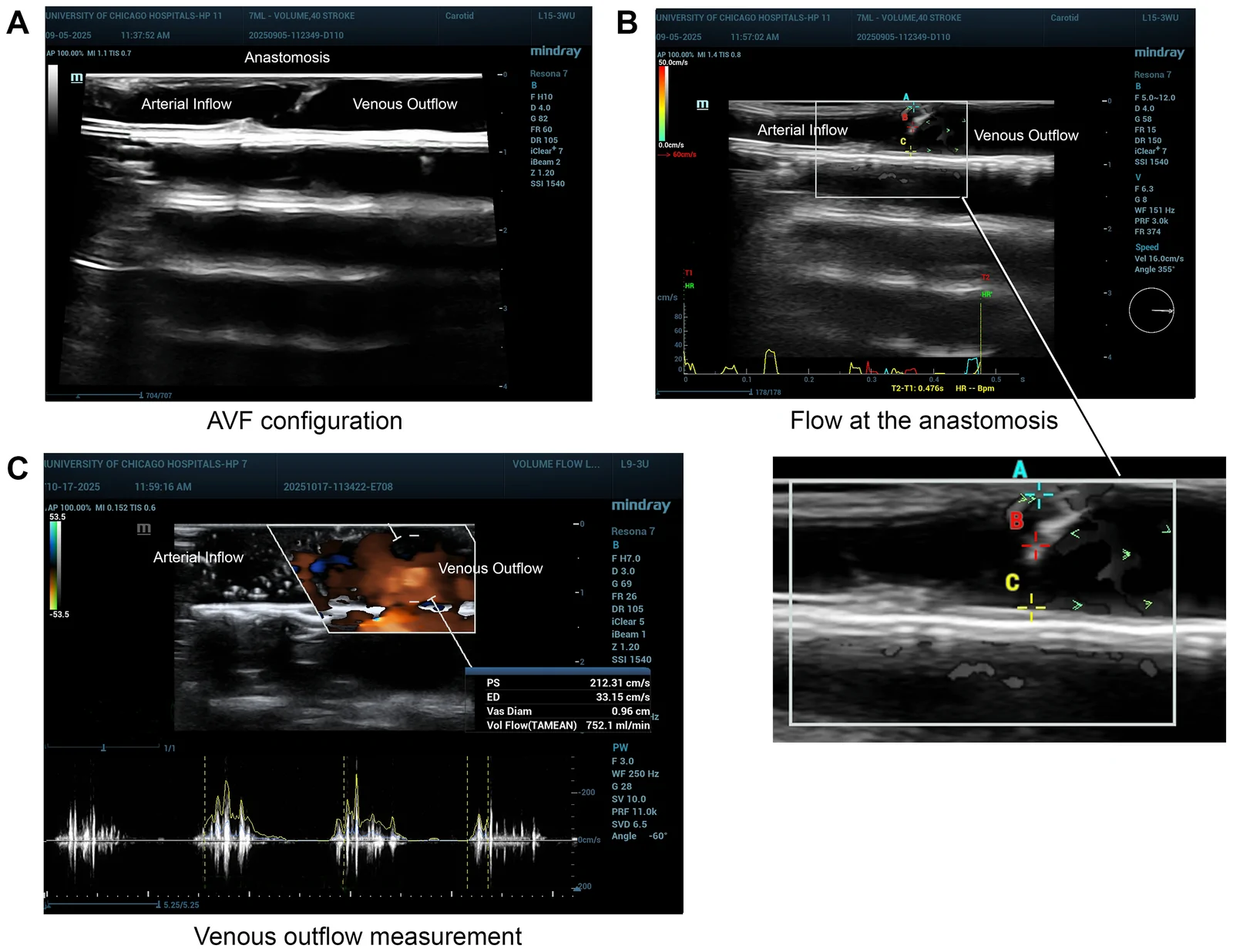
Doppler ultrasound assessment of flow in the surgically constructed ex vivo arteriovenous fistula (*AV*F). Representative ultrasound images were acquired with L15-3WU and L9-3U linear-array transducers. **A,** B-mode ultrasound image showing the arterial inflow, anastomosis, and venous outflow. **B,** Doppler ultrasound image demonstrating flow at the anastomosis; directional arrows indicate local flow direction, with disordered orientation consistent with turbulent AVF hemodynamics. **C,** Spectral Doppler assessment of the venous outflow demonstrated a volume flow rate of 752.1 mL/min during perfusion at 40 strokes/min with a 7 mL stroke volume.

Histologic evaluation of postperfusion AVF tissue demonstrated that the constructed AVF remained intact and had clear arterial and venous components and close tissue apposition at the anastomosis. Hematoxylin and eosin staining demonstrated distinct arterial and venous segments (Fig 4, *A*). Masson trichrome staining highlighted collagen-rich extracellular matrix in blue and vascular smooth muscle/cytoplasmic elements in red, supporting structural identification of the arterial and venous components of the AVF (Fig 4, *B*).

**Fig 4:**
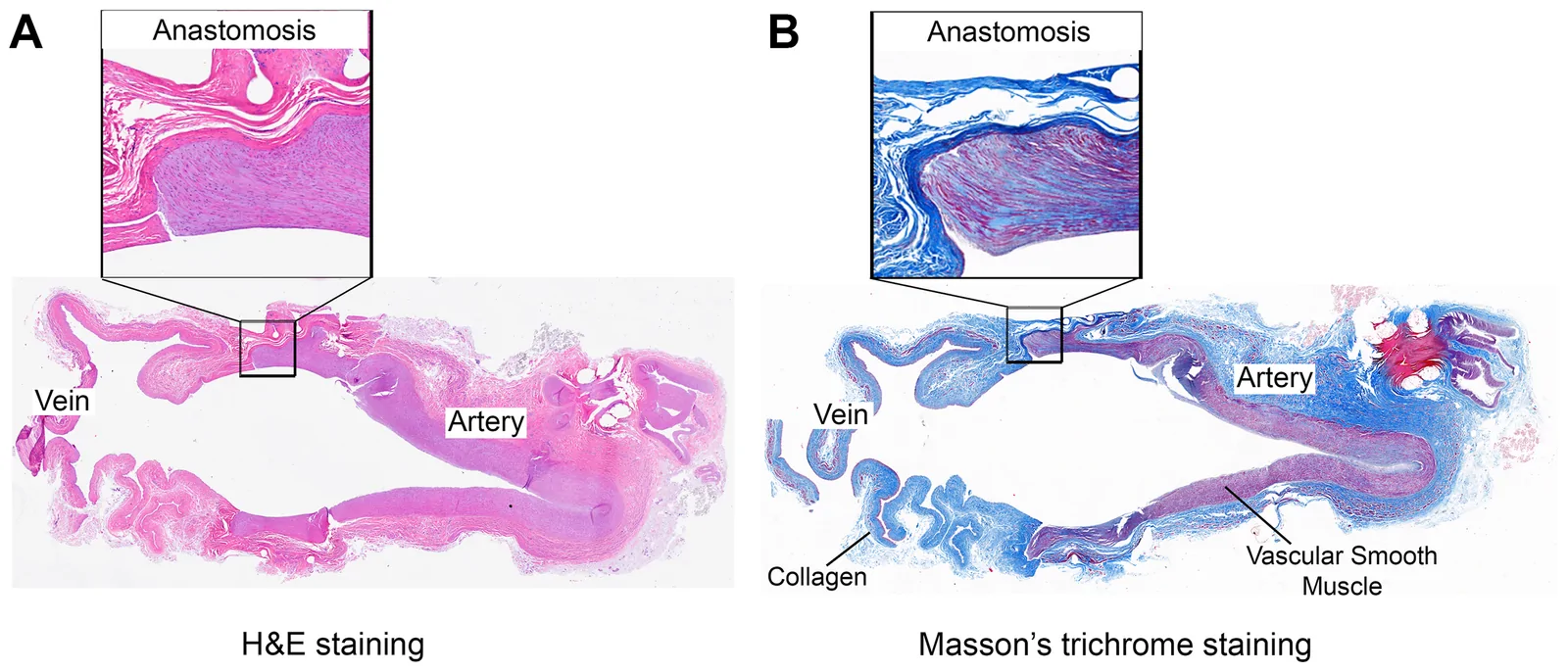
Histologic architecture of the surgically constructed human iliac arteriovenous fistula (AVF). **A,** Hematoxylin and eosin (*H&E*) staining demonstrates distinct arterial and venous segments with close tissue apposition at the anastomosis. **B,** Masson trichrome staining highlights collagen-rich extracellular matrix in blue and vascular smooth muscle/cytoplasmic elements in red, supporting structural identification of the arterial and venous components of the AVF. Insets show the anastomotic interface. Original magnification, ×10.

### Apoptosis assessment of vessel viability and tissue age

Pre- and postperfusion iliac AVF artery, AVF vein, and straight artery samples were initially evaluated by TUNEL staining and Fiji (ImageJ) with Otsu automated thresholding. Whole-section TUNEL-positive area did not increase after perfusion, measuring 10% preperfusion and 5.5% postperfusion in AVF arteries, 14% and 1% in AVF veins, and 20% and 13.3% in straight arteries, respectively (Fig 5).

**Fig 5:**
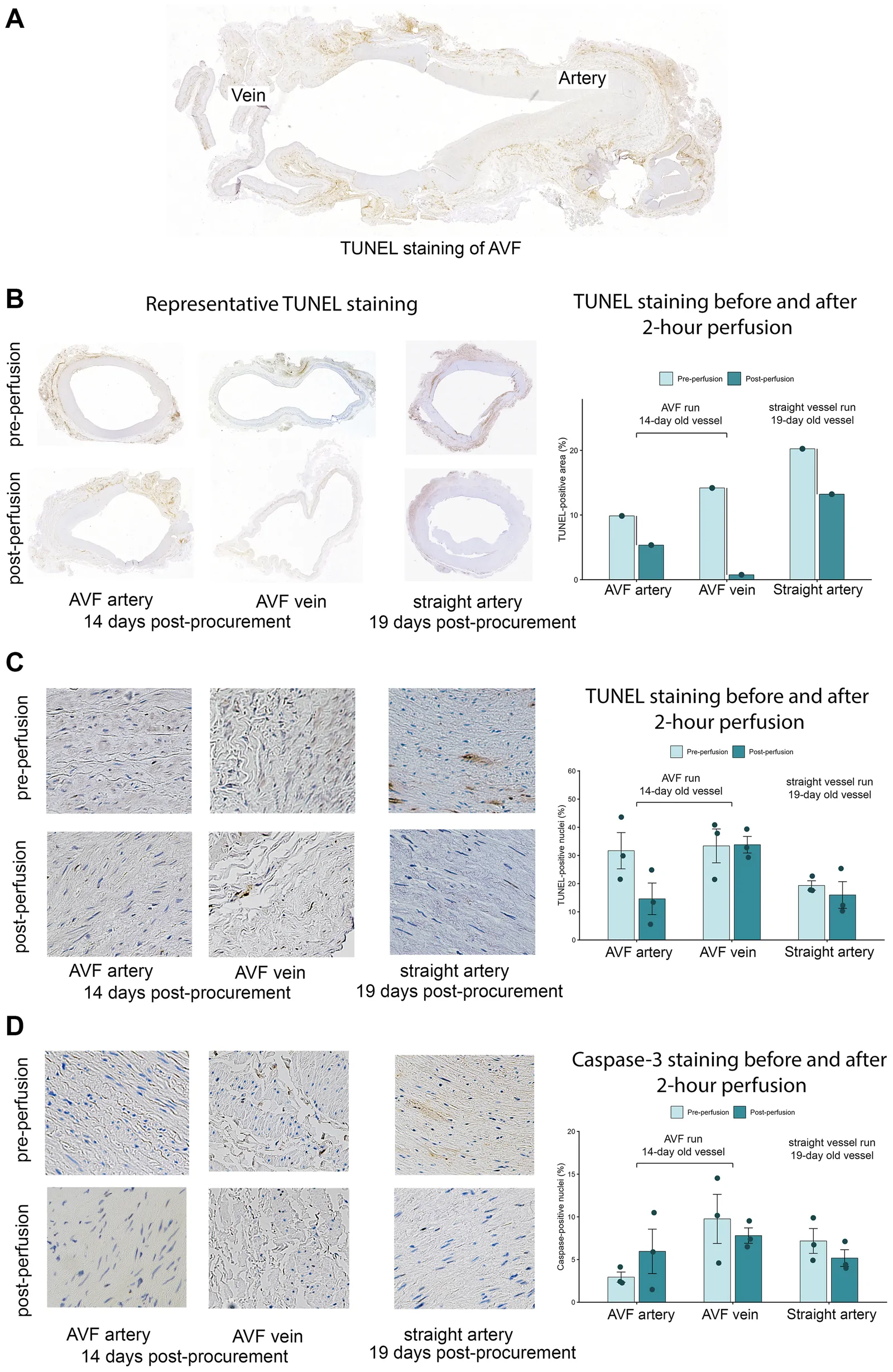
Cleaved caspase-3 and terminal deoxynucleotidyl transferase dUTP nick-end labeling (*TUNEL*) staining support maintenance of human iliac vessel viability during ex vivo pump perfusion. **A,** Representative TUNEL-stained cross-section of a surgically constructed arteriovenous fistula (*AVF*), showing the vein and artery in continuity. Magnification, ×10. **B,** Representative TUNEL-stained sections (*left*) and quantification of TUNEL-positive area (*right*) before and after 2-hour ex vivo perfusion. Columns show AVF artery and AVF vein from the same 14-day AVF run, and straight artery from a separate 19-day straight-vessel run. Magnification, ×10. Quantification performed using Otsu thresholding in Fiji (ImageJ). **C,** TUNEL-stained sections (*left*) and quantification of TUNEL-positive nuclei (*right*) before and after 2-hour perfusion, expressed as a percentage of total nuclei across three random sections per sample. Magnification, ×20. **D,** Cleaved caspase-3-stained sections (*left*) and quantification of cleaved caspase-3-positive nuclei (*right*) before and after 2-hour perfusion, expressed as a percentage of total nuclei across three random sections per sample. Magnification, ×20.

To validate the whole-section analysis, three randomly selected ×20 fields of view were analyzed from each specimen, and TUNEL-positive and total nuclei were manually counted. The same sampling strategy was applied to adjacent sections stained for cleaved caspase-3 immunohistochemistry. Manual quantification demonstrated no evidence of increased apoptosis after perfusion. The percentage of TUNEL-positive nuclei in AVF arteries decreased from 31.69% ± 6.43% preperfusion to 14.61% ± 5.60% postperfusion, remained similar in AVF veins (33.40% ± 6.01% vs 33.82% ± 2.95%), and decreased modestly in straight arteries (19.35% ± 1.66% vs 15.98% ± 4.73%). TUNEL-positive nuclei were consistently more abundant than cleaved caspase-3-positive nuclei, consistent with TUNEL detection of DNA fragmentation arising from both apoptotic and nonapoptotic processes. Still, the percentage of cleaved caspase-3-positive nuclei remained stable following perfusion. In AVF arteries, cleaved caspase-3-positive nuclei measured 2.94% ± 0.60% preperfusion and 5.96% ± 2.60% in postperfusion. In AVF veins, values were 9.76% ± 2.88% and 7.80% ± 0.90%, respectively. In straight arteries, values were 7.18% ± 1.45% and 5.17% ± 0.98%, respectively. Together, these findings are consistent with the whole-section TUNEL analysis and do not indicate an increase in apoptotic injury during short-term ex vivo perfusion (Fig 5).

Representative iliac arteries and veins were also fixed and TUNEL-stained at 3, 7, 14, and 26 days after vessel acquisition. Fiji Otsu threshold quantification showed that TUNEL-positive area increased with vessel age in both arteries and veins. In arteries, staining increased from 1.74% at day 3 to 9.86% at day 14 and 48.70% at day 26, representing 5.7-fold and 28.0-fold increases from day 3, respectively. In veins, staining increased from 1.37% at day 3 to 14.19% at day 14 and 34.86% at day 26, representing 10.3-fold and 25.4-fold increases from day 3, respectively (Fig 6).

**Fig 6:**
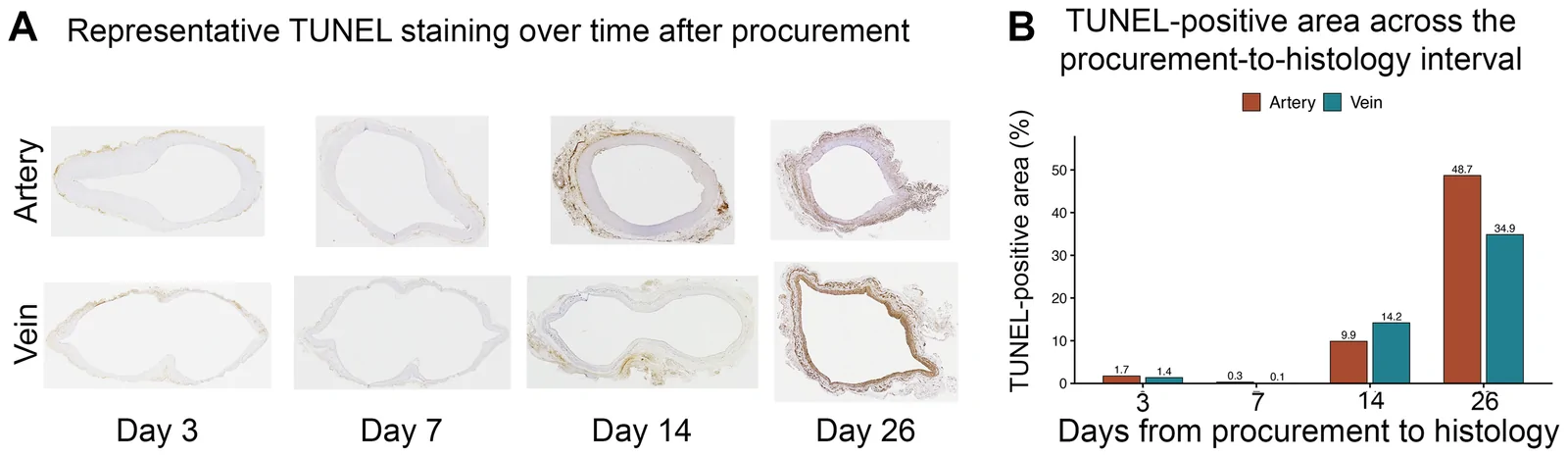
Terminal deoxynucleotidyl transferase dUTP nick (*TUNEL*) staining of ex vivo human iliac vessels across the procurement-to-histology interval. **A,** Representative TUNEL-stained sections of human iliac arteries and veins fixed 3, 7, 14, or 26 days after procurement. Images were selected to illustrate representative staining quality across the procurement-to-histology interval. Magnification ×10. **B,** TUNEL-positive area (%) measured using Fiji with Otsu automatic thresholding for each vessel across the procurement-to-histology interval. n = 1 vessel per time point.

## Discussion

We developed a modular ex vivo adult human iliac vessel perfusion platform that enables controlled study of acute vascular tissue responses to mechanical, biochemical, and device-related interventions across multiple vascular configurations and experimental exposures. As a key proof of concept, this platform supported surgical construction of a human AVF using standard end-to-side anastomotic technique, followed by pump perfusion, Doppler ultrasound assessment, and histologic analysis.

Previous ex vivo vascular platforms have demonstrated the value of controlled vessel-based systems across several contexts, including the analysis of aortic pathology.^10^, ^11^, ^12^ Our platform builds on this foundation by using discarded adult human iliac arteries and veins in a modular perfusion system that supports surgical manipulation and multiple vascular configurations. Unlike platforms designed for a single experimental application, the modular design permits adaptation to a variety of clinically relevant vascular interventions, including device deployment, therapeutic delivery, and surgically reconstructed vascular configurations. The platform is particularly well-suited for studies of acute vascular responses, evaluation of vascular-targeted therapeutics under physiologic flow, and preclinical assessment of endovascular devices or surgical techniques in viable human vessels. By enabling controlled perfusion of intact human vessels with surgical manipulation and hemodynamic assessment, this platform can help address unanswered questions regarding how acute hemodynamic changes influence early endothelial and smooth muscle cell responses in human vessels, how vessel age and procurement conditions affect tissue suitability for experimentation, and how device deployment or therapeutic delivery alters vascular biology under physiologic flow in human tissue.

The AVF configuration was selected as a key application because of its surgical complexity, clinical relevance, and dependence on local hemodynamic conditions.^13^ AVF maturation and failure remain major clinical challenges, and failure of fistula maturation or long-term patency continues to limit durable vascular access.^14^ In this study, the surgically constructed ex vivo AVF achieved venous outflow volume-flow rates above the historical Kidney Disease Outcomes Quality Initiative “Rule of 6s” threshold of 600 mL/min, supporting the physiologic plausibility of the model.^15^ The presence of disturbed flow at the anastomosis further supports the ability of the platform to reproduce key qualitative features of AVF hemodynamics.

The pre- and postperfusion TUNEL findings suggest that short-term pump perfusion did not measurably increase apoptotic burden in the evaluated vessels. The higher proportion of TUNEL-positive nuclei relative to cleaved caspase-3-positive nuclei is consistent with the distinct processes detected by these assays: TUNEL identifies DNA fragmentation, which may arise during apoptosis or other forms of cellular injury, whereas cleaved caspase-3 specifically marks caspase-dependent apoptosis. Manual quantification of apoptotic nuclei in randomly selected ×20 fields and confirmatory cleaved caspase-3 immunohistochemistry produced consistent results, supporting the conclusion that short-term perfusion did not increase apoptosis. The observed postperfusion decreases in TUNEL-positive area should be interpreted cautiously and may reflect cell loss during perfusion rather than a protective effect of the pump circuit.

Although AVF maturation is a normal adaptive process, neointimal hyperplasia is a pathologic process that contributes to AVF failure. Apoptosis is an integral component of vascular cell turnover that maintains vascular homeostasis; when the balance between proliferation and apoptosis is disrupted, excessive neointimal growth ensues.^16^ While these roles of apoptosis in vascular remodeling are well established, the 2-hour perfusion interval in the present study was designed to assess acute tissue viability rather than longer-term remodeling processes such as AVF maturation or neointimal hyperplasia. Accordingly, within this experimental timeframe, TUNEL and cleaved caspase-3 staining are interpreted primarily as markers of perfusion-related injury rather than remodeling processes. Within this acute experimental window, the platform is better suited to investigations of early vascular responses, including transcriptional, inflammatory, and signaling changes following controlled perfusion.

Separately, vessel-age histology demonstrated an increase in TUNEL-positive area with longer time from acquisition to fixation, supporting the importance of vessel age as a quality-control variable in future experiments. These findings are consistent with previous studies demonstrating progressive loss of cell viability and increasing apoptotic burden with prolonged storage of preserved human iliac vessels.^17^ Although progressive loss of tissue viability with prolonged storage is expected, this analysis was included specifically to define the practical time window over which discarded transplant vessels remain suitable for acute ex vivo experimentation. These findings should be interpreted cautiously because the number of available vessels was limited, and vessel availability depended on transplant workflow and discard timing.

Several limitations should be noted. First, the platform used endothelial cell culture medium supplemented with 1× penicillin-streptomycin rather than whole blood. This limits direct modeling of hematologic-endothelial interactions, thrombosis, circulating inflammatory cells, and blood viscosity effects, although it improved controllability and sterility for this proof-of-concept platform. Accordingly, diastolic pressures were subphysiologic, an expected consequence of the lower viscosity of endothelial cell culture medium relative to whole blood; substitution of more biologic fluids represents a promising direction for future work. Second, the experiments were performed over a short perfusion interval and therefore do not model longer-term remodeling or maturation. Third, the number of AVF runs was limited, and ultrasound and pressure measurements were not available uniformly across all experiments. Fourth, as vessels were deidentified discarded transplant vessels, donor-level clinical characteristics were not available. Finally, the use of iliac vessels reflects the availability of discarded transplant tissue and does not fully recapitulate the caliber of upper-extremity vessels typically used for hemodialysis access. This caliber difference may limit direct translation of the observed hemodynamic and remodeling responses to clinical AVF physiology, as wall shear stress, flow dynamics, and anastomotic behavior vary with vessel size. Nevertheless, the platform’s modular design could be adapted to smaller vessels as appropriate tissue becomes available.

## Conclusions

In summary, we developed a modular ex vivo human vessel perfusion platform capable of supporting pulsatile flow and surgically constructed AVF configurations with clinically relevant flow conditions. By combining controlled perfusion, Doppler ultrasound assessment, and histologic analysis, this system provides a biologically viable human tissue platform for studying mechanical, biochemical, and device-related contributors to vascular reconstruction. Future applications include nanoparticle therapeutic delivery, stent apposition assessment, anastomotic configuration analysis and repair, and investigation of acute vascular histological responses in human tissue.

## Author Contributions

Conception and design: CW, MW, KN, NN, PS, LP, SG

Analysis and interpretation: CW, MW, KN, MG, SK, NN, LP, SG

Data collection: CW, MW, MG, SK, IK

Writing the article: CW

Critical revision of the article: CW, MW, KN, MG, SK, NN, IK, PS, LP, SG

Final approval of the article: CW, MW, KN, MG, SK, NN, IK, PS, LP, SG

Statistical analysis: Not applicable

Obtained funding: Not applicable

Overall responsibility: CW

CTW and MW contributed equally to this work and share first authorship.

## Funding

This work was supported by the Department of Surgery at the 10.13039/100007234University of Chicago. The Department of Surgery had no involvement in the study design; collection, analysis, or interpretation of data; manuscript writing; the decision to submit the manuscript for publication; or any other aspect of manuscript preparation.

## Declaration of generative AI and AI-assisted technologies in the writing process

During the preparation of this work, the author used the Perplexity AI assistant to assist with the grammar and language editing. After using this tool, the authors reviewed and edited the content as needed and take full responsibility for the content of the publication.

## Disclosures

The authors declare no conflicts of interest related to this work.

## References

[1] Korshunov V.A., Schwartz S.M., Berk B.C. (2007). Vascular remodeling: hemodynamic and biochemical mechanisms underlying Glagov’s phenomenon. Arterioscler Thromb Vasc Biol.

[2] Huber T.S., Berceli S.A., Scali S.T. (2021). Arteriovenous fistula maturation, functional patency, and intervention rates. JAMA Surg.

[3] Méndez-Barbero N., Gutiérrez-Muñoz C., Blanco-Colio L.M. (2021). Cellular crosstalk between endothelial and smooth muscle cells in vascular wall remodeling. Int J Mol Sci.

[4] Kural M.H., Dai G., Niklason L.E., Gui L. (2018). An ex vivo vessel injury model to study remodeling. Cell Transplant.

[5] Al-Sowayan B., Keogh R.J., Abumaree M., Georgiou H.M., Kalionis B. (2019). An ex vivo human placental vessel perfusion method to study mesenchymal stem/stromal cell migration. Stem Cell Investig.

[6] Bianco R., Di Gregoli K., Caputo M., George S.J., Johnson J.L. (2020). A protocol for a novel human ex vivo model of aneurysm. STAR Protoc.

[7] Lysyy T., Bracaglia L.G., Qin L. (2020). Ex vivo isolated human vessel perfusion system for the design and assessment of nanomedicines targeted to the endothelium. Bioeng Transl Med.

[8] Atigh M.K., Goel E., Erwin M. (2021). Precision delivery of liquid therapy into the arterial wall for the treatment of peripheral arterial disease. Sci Rep.

[9] OPTN/UNOS (2018).

[10] Faure E.M., Canaud L., Cathala P., Serres I., Marty-Ané C., Alric P. (2014). Human ex vivo model of Stanford type B aortic dissection. J Vasc Surg.

[11] Dziodzio T., Juraszek A., Reineke D. (2011). Experimental acute type B aortic dissection: different sites of primary entry tears cause different ways of propagation. Ann Thorac Surg.

[12] Laffey M., Tornifoglio B., Lally C. (2023). Development and initial characterisation of a localised elastin degradation ex vivo porcine aortic aneurysm model. Appl Sci.

[13] Browne L.D., Bashar K., Griffin P., Kavanagh E.G., Walsh S.R., Walsh M.T. (2015). The role of shear stress in arteriovenous fistula maturation and failure: a systematic review. PLoS One.

[14] Lee T., Chauhan V., Krishnamoorthy M. (2011). Severe venous neointimal hyperplasia prior to dialysis access surgery. Nephrol Dial Transplant.

[15] National Kidney Foundation (2006). KDOQI clinical practice guidelines for vascular access: guideline 3. https://kidneyfoundation.cachefly.net/professionals/KDOQI/guideline_upHD_PD_VA/va_guide3.htm.

[16] Korshunov V.A., Berk B.C. (2008). Smooth muscle apoptosis and vascular remodeling. Curr Opin Hematol.

[17] Ren Z.Y., Pan B., Wang F.F., Lyu S.C., He Q. (2023). Effects of different preservation methods of human iliac veins. Cell Tissue Bank.

